# Phytoremediation-agrivoltaic systems for PTEs decontamination: a case study from an industrial site in Augusta, Italy

**DOI:** 10.1007/s11356-026-38149-1

**Published:** 2026-09-02

**Authors:** Michele Di Agosto, Claudio Armaro, Samuele Di Novo, Francesco Salmeri, Nico Randazzo, Francesco Sergi

**Affiliations:** 1 ITAE CNR: Istituto Di Tecnologie Avanzate Per L’Energia Nicola Giordano Consiglio Nazionale Delle Ricerche, Contrada Bufalaro, Augusta, SR Italy; 2 ITAE CNR: Istituto Di Tecnologie Avanzate Per L’Energia Nicola Giordano Consiglio Nazionale Delle Ricerche, Consiglio Nazionale delle Ricerche, Messina, Italy

**Keywords:** Pollution, Decontamination, Potentially toxic elements (PTEs), Phytoremediation, Agrivoltaic, Photovoltaic

## Abstract

Over the past century, industrial activities have caused widespread soil contamination by potentially toxic elements (PTEs), making effective remediation increasingly urgent. Phytoremediation offers a sustainable strategy using selected plant species to remove, stabilize, or sequester pollutants. This study proposes an integrated agrivoltaic-phytoremediation framework for remediating PTE-contaminated soils. The framework integrates literature-based plant selection, photovoltaic system simulations, and techno-economic assessment, demonstrated through a representative case study in Augusta (Sicily, Italy). The effects of photovoltaic shading on C3 and C4 crops, together with PTE contamination, were estimated from literature data to quantify biomass reduction. A quantitative assessment was then performed by integrating literature data with PVsyst simulations of different photovoltaic configurations. The results indicate that the optimal configuration depends on the specific system objectives, such as maximizing energy production or biomass yield. Three agrivoltaic configurations, each combining a different photovoltaic technology with a selected plant species, were evaluated through a techno-economic assessment considering energy production, biomass yield under varying shading conditions, and net economic returns. The monofacial/*Arundo donax* L., bifacial/*Chrysopogon zizanioides* (L.) Roberty, and semi-transparent/*Cannabis sativa* L. configurations achieved annual energy productions of 568,660, 297,987, and 285,638 kWh, respectively, with corresponding biomass yields of 26.3, 60.0, and 14.0 t ha^−1^ year^−1^ and net economic returns of €2,906, €6,730, and €1,543 ha^−1^ year^−1^.

## Introduction

Soil contamination by potentially toxic elements (PTEs) has become a major global concern. Currently, more than 10 million sites worldwide are estimated to be contaminated (Li et al. 2022; Luo et al. 2023). Major sources of pollution include mining activities, foundries, industrial waste, petrochemical, and electronics production, as well as fossil fuel combustion (Fry et al. 2020; Khan et al. 2021; Schneider et al. 2016). Although fossil fuels remain essential for chemical manufacturing and electricity generation, their widespread use, particularly in internal combustion engines, significantly contributes to environmental degradation through emissions of CO₂, nitrogen oxides, sulfur dioxide, volatile organic compounds, and PTEs. Urban expansion and intensive fertilizer use (Cherniwchan 2012; Mózner et al. 2012) have further reduced arable land, while the increasing demand for raw materials and energy continues to exacerbate the problem (Kamran et al. 2021; Saleem et al. 2020; Ali et al. 2020). Immediate remediation actions are therefore required. The European Green Deal and the EU Biodiversity Strategy for 2030 emphasize the importance of protecting soil fertility, reducing erosion, and increasing organic matter content through sustainable soil management practices.

Traditionally, contaminated soils have often been excavated and disposed of in landfills (Sharma et al. 2018). In recent years, phytoremediation has emerged as a sustainable alternative, exploiting plants to sequester, degrade, or stabilize pollutants in soil and water (Putra et al. 2025; Tiwari et al. 2025; Rahman et al. 2024; Kafle et al. 2022; Ashraf et al. 2019). Its effectiveness depends on the plant species and the type of contaminant (Luo and Zhang 2021; Madhav et al. 2024; Mahar et al. 2016). Some hyperaccumulator plants can absorb large amounts of PTEs without suffering physiological damage (Liu et al. 2025). Suitable species are characterized by high biomass yield, pollutant tolerance, ease of cultivation, and low palatability to herbivores (Adesodun et al. 2010; Ha et al. 2011; Shabani and Sayadi 2012). This study explores the integration of phytoremediation with agrivoltaics, combining photovoltaic energy generation with biomass yield on the same land (Leon and Ishihara 2018; Liu et al. 2018). In addition to improving land-use efficiency, this integration directly contributes to soil remediation, addressing environmental degradation while simultaneously producing renewable energy. Agrivoltaics can also improve the economic viability of phytoremediation, promoting its application on marginal or contaminated lands that can thereby be reclaimed and returned to productive use.

Another significant advantage of the proposed approach is the possibility of valorizing the harvested biomass through energy and industrial applications, further improving the economic sustainability of phytoremediation. In addition, agrivoltaic systems may enhance crop resilience by reducing evapotranspiration and providing controlled shading while simultaneously generating renewable electricity (Sgroi et al. 2014; Calvert and Mabee 2015).

The main objective of this study is to develop and evaluate an integrated agrivoltaic-phytoremediation framework capable of simultaneously supporting soil remediation, biomass production, and renewable energy generation in PTE-contaminated areas. The proposed methodology combines a literature-based selection of plant species and photovoltaic technologies with PVsyst simulations and a techno-economic assessment. The industrial area of Augusta (Sicily, Italy) is adopted as a representative case study to demonstrate the applicability of the proposed framework under realistic environmental conditions.

## Materials and methods

### Literature survey

This study developed and evaluated an integrated agrivoltaic-phytoremediation framework for the sustainable management of PTE-contaminated soils. The objective was to identify the most suitable combinations of plant species, contaminated soil conditions, photovoltaic shading configurations, and geographical settings to maximize both remediation efficiency and renewable energy production. The industrial area of Augusta (Sicily, Italy), one of the most heavily contaminated industrial sites in Italy, was selected as a representative case study to evaluate the applicability of the proposed integrated approach under realistic environmental conditions. The study was based on information collected from the scientific literature, site-specific data from the Augusta case study, and simulations performed using PVsyst together with spreadsheet-based analyses. Relevant scientific literature was identified through searches conducted in the Scopus, Web of Science, and Google Scholar databases, focusing on phytoremediation of PTE-contaminated soils, agrivoltaic systems, biomass production under contaminated conditions, crop responses to photovoltaic shading, and biomass valorization pathways.

Preliminary searches using broad terms such as “phytoremediation,” “decontamination,” “potentially toxic elements (PTEs),” “agrivoltaic,” and “photovoltaic” returned several thousand records, reflecting the breadth of the available scientific literature. To ensure consistency with the objectives of the study, the search strategy was progressively refined through combinations of targeted keywords and Boolean operators.

Representative search strings included: “phytoremediation AND potentially toxic elements,” “phytoremediation AND contaminated soils,” “agrivoltaic AND biomass production,” “photovoltaic shading AND crop yield,” “phytoremediation AND biomass valorization,” as well as searches including the scientific names of the selected species (*Cannabis sativa* L., *Chrysopogon zizanioides* (L.) Roberty, and *Arundo donax* L.). Additional relevant publications were identified through the reference lists of review papers and research articles.

Priority was given to studies conducted in Mediterranean, semi-arid, or industrially contaminated areas characterized by environmental conditions comparable to those of Augusta. Priority was given to peer-reviewed studies providing quantitative information on plant tolerance to PTEs, biomass production, phytoremediation performance, photovoltaic shading effects, and biomass valorization pathways. Following title, abstract, and full-text screening, approximately 100 publications were retained.

The collected information was subsequently used to define the selection criteria for plant species and photovoltaic technologies and to support the development and evaluation of the integrated agrivoltaic-phytoremediation framework proposed in this study. Owing to the heterogeneity of the available studies, the literature survey provided the methodological basis for plant selection, photovoltaic system design, and numerical simulations rather than constituting a systematic review or meta-analysis.

### Methodology for phytoremediation species selection

Plant species were selected according to phytoremediation criteria identified from the scientific literature. The selection process considered the characteristics of contaminated sites, pollutant properties, plant physiological traits, remediation mechanisms, biomass productivity, and the potential for biomass valorization (Ashraf et al. 2019). Accurate site characterization is essential for selecting the most appropriate phytoremediation strategy. A contaminated site is typically defined as an area where ongoing or past human activities have significantly altered the natural characteristics of environmental matrices (Aziz and Mujeeb 2022; Wuana and Okieimen 2011). Table 1 shows the main PTEs present in the polluted sites and their sources (Ali et al. 2013).

**Table 1:** Main anthropogenic sources of potentially toxic elements (PTEs) commonly detected in contaminated soils. The table summarizes the principal industrial, agricultural, and urban activities responsible for the release of individual PTEs into the environment, providing the background information used to identify the contaminants of interest in phytoremediation studies and in the Augusta case study. Adapted from Aswal et al. (2023)

PTEs	Sources	Reference
Al	Application as a coagulant and mineral weathering of feldspars	Prasad et al. (2022)
Cr	Effluent, tanning, electroplating, and pigment production	Vidu et al. (2020)
Mn	Mn-containing agrochemicals, municipal wastewater, and sewage sludge	Röllin and Nogueira (2011)
Cu	Pesticides, fertilizers, corrosion of distribution pipes and erosion from natural deposits	Obasi and Akudinobi (2020)
Zn	rock weathering, industrial and domestic wastewater	Obasi and Akudinobi (2020)
As	Industrial wastes, metallic wastes, etc.	Mohammed Abdul et al. (2015)
Se	Compounds have dominance of silver, sulfur, copper, lead and nickel	Obasi and Akudinobi (2020)
Cd	phosphate fertilizers, and waste incineration	Genchi et al. (2020)
Pb	Lead-based batteries, solder, alloys, rust inhibitors, and plastic stabilizers	World Health Organization (2017)

The preliminary characterization of contaminated sites was based on the identification of the main contaminants, their concentrations, and the environmental conditions affecting phytoremediation performance. When referring to the Augusta case study, contamination levels and site characteristics were derived from published literature and interpreted according to the Italian environmental legislation (Legislative Decree 152/2006). Several key factors must be considered before evaluating the choice of intervention through phytoremediation. These factors help to determine the most suitable approach.

The first step is to characterize the physical and chemical properties of the contaminant, including its spatial distribution and concentration. Specifically, it is important to determine the distribution of the contaminant among its different phases (free, adsorbed, dissolved, and gaseous). Additionally, characteristics such as toxicity, flammability, explosiveness, stability, mobility, persistence, and biodegradability should be identified to select the most effective phytoremediation mechanism (Guarino et al. 2019).

Accordingly, the selection criteria also included geological, hydrogeological, climatic, and land-use characteristics, since these factors strongly influence plant adaptation and the overall feasibility of phytoremediation interventions.

Based on these criteria, phytoremediation was selected as the remediation strategy because of its compatibility with contaminated agricultural soils, low environmental impact, and potential integration with agrivoltaic systems. Technical feasibility, site characteristics, biomass productivity, and long-term sustainability were considered during the evaluation process (Santos et al. 2025; Alsafran et al. 2023).

The classification of plants used for phytoremediation is essential for optimizing environmental decontamination efforts. The species employed can be divided into perennial and annual plants, each with specific characteristics that influence their effectiveness. Perennial plants, due to their longevity and deep root system, are ideal for long-term interventions, while annual species, with their rapid life cycle, offer an immediate solution for the removal of surface contaminants (Subpiramaniyam 2021). Additionally, the choice of species depends on the type of contaminant and the application environment: herbaceous species are distinguished by their rapid accumulation capacity, while woody plants can sequester larger amounts of pollutants due to their high biomass (Bian et al. 2020).

An initial screening of the literature identified several perennial species previously investigated for the phytoremediation of PTE-contaminated soils. Representative examples are reported in Table 2 and are used as the starting point for the subsequent selection of candidate species (Poppenwimer et al. 2023).

**Table 2:** Representative perennial plant species investigated for the phytoremediation of potentially toxic element (PTE)-contaminated soils. The table reports the main perennial species identified in the literature, the PTEs targeted by each species, and the corresponding references. These species were used as the initial candidate pool for the selection process adopted in this study

Plants	PTEs	Reference
*Chrysopogon zizanioides* (L.) Roberty	Fe Cu Zn Cr Mn Pb	Banerjee et al. (2016)
*Arundo donax* L.	Zn Cr Pb	Barbosa et al. (2015)
*Miscanthus sinensis* Anderson	Zn	Barbosa et al. (2015)
*Triticum aestivum* L.	Zn Cd	Chaplygin et al. (2020)
*Phragmites australis* (Cav.Trin.exsteud)	Cd Mn Cr Zn Cu As Pb Ni	Rezania et al. (2019)

A similar screening was performed for annual species with documented phytoremediation potential. Representative annual species are reported in Table 3 and are subsequently evaluated together with perennial species according to the selection criteria adopted in this study (Poppenwimer et al. 2023).

**Table 3:** Representative annual plant species reported in the literature for the phytoremediation of potentially toxic element (PTE)-contaminated soils. The table lists the principal annual species, the target PTEs reported in previous studies, and the corresponding literature sources. These species were comparatively assessed together with perennial species during the selection process for the proposed agrivoltaic-phytoremediation framework

Plants	PTEs	Reference
*Sorghum bicolor* (L.) Moench	Ni Pb Zn	Al Chami et al. (2015)
*Carthamus tinctorius* L.	Ni Pb Zn	Al Chami et al. (2015)
*Helianthus annuus* L.	As Cd Cr Pb Zn Cu Ni	Monisha and Sangeetha (2025)
*Cannabis sativa* L.	Cd Pb Cr Ni Zn Cu	Jurga et al. (2025)

The final selection of candidate species considered the main factors influencing phytoremediation efficiency reported in the scientific literature. These included contaminant bioavailability, biomass productivity, root system development, tolerance to potentially toxic elements (PTEs), and the predominant remediation mechanism adopted by each species (Kafle et al. 2022). Since plants can absorb contaminants only when they are present in bioavailable forms, their bioavailability was considered a key selection criterion for evaluating the suitability of phytoextraction as a remediation strategy (Petruzzelli et al. 2015). The literature also reports that the application of chelating agents may enhance contaminant bioavailability and improve phytoextraction efficiency under specific conditions; however, no chelating agents were considered in the present study (Dipu et al. 2012). Additional environmental factors known to influence phytoremediation performance, including soil pH, electrical conductivity, organic matter content, and microbial activity, were also considered because of their effects on contaminant mobility and plant uptake (Lay et al. 2025; Guidi Nissim et al. 2018). Furthermore, the contribution of plant-associated microorganisms to contaminant degradation, detoxification, immobilization, and plant growth promotion was considered during the evaluation of the selected species (Shen et al. 2022; Weyens et al. 2015; Gomes et al. 2016; Falfushynska et al. 2024; Lee et al. 2020). The phytoremediation potential of the candidate species was subsequently evaluated using the most widely adopted performance indicators reported in the scientific literature:

- Bioconcentration factor or bioaccumulation factor (BCF or BAF): the ability of the plant to absorb metals contained in the soil; it is given by the ratio between the concentration of metals in the plant (referred to the dry weight) and the mobile soil fraction and it is useful for comparing different hyperaccumulators (Aziz and Mujeeb 2022).
- Bioaccumulation coefficient: the ratio between the metal concentration in the plant (g metal/g plant dry weight) and the initial concentration of the metal in solution in the mobile soil fraction (mg metal/L) (Hidayati and Setyo Rini 2020).
- Remediation capacity or remediation factor: average quantity of elements extracted from plants compared to the total concentration of elements in the soil for a growing season; it is obtained by dividing the total quantity of elements present in the plant biomass by the initial total concentration of contaminants in the soil, compared to the volume of soil considered (Hidayati and Setyo Rini 2020).
- Translocation factor (TF): the ability of the plant to transfer metals from the roots to the upper parts of the stem and into the leaves; it is obtained by dividing the concentration of the metal in the leaves and roots. A species that has TF > 1 is therefore defined as a translocator. On the opposite side, decreasing this parameter increases the adaptability of the plant to phytostabilization techniques (Hidayati and Setyo Rini 2020).

### Criteria for photovoltaic system selection

Photovoltaic technologies were selected according to criteria relevant to their integration with phytoremediation systems. The evaluation considered energy production, shading characteristics, compatibility with crop growth, land-use efficiency, and adaptability to the environmental conditions of the Augusta case study. Emphasis was placed on photovoltaic configurations capable of improving agricultural resilience through controlled shading while maximizing renewable electricity generation and maintaining adequate biomass production on the same land (Agostini et al. 2021; Klokov et al. 2023; Zahrawi and Aly 2024).

The proposed agrivoltaic configurations were evaluated considering the current Italian regulatory framework governing the installation of photovoltaic systems on agricultural and contaminated land. Priority was given to the environmental legislation regulating contaminated sites, the authorization procedures for photovoltaic installations, and the most recent national provisions promoting the integration of renewable energy production with agricultural and environmental remediation activities. The proposed configurations were therefore developed to be consistent with the current Italian legislative and regulatory framework applicable to agrivoltaic systems.

The photovoltaic layout was designed to minimize the impact of shading on crop growth while maintaining high electricity production. The evaluation considered row spacing, module orientation, tilt angle, and the resulting distribution of photosynthetically active radiation (PAR), since these parameters directly influence both biomass production and photovoltaic performance (Barbosa et al. 2015). The photovoltaic configuration was subsequently assessed considering crop characteristics, local climatic conditions, and the technical requirements of the photovoltaic system (Table 4).

**Table 4:** Main factors affecting the design and performance of agrivoltaic systems. The table summarizes the principal parameters influencing both photovoltaic electricity generation and crop development, highlighting the trade-offs between energy production and agricultural productivity. These criteria were used to support the selection of the photovoltaic configurations evaluated in the proposed agrivoltaic-phytoremediation framework (Barron-Gafford et al. 2019; Gomez-Casanovas et al. 2023)

Issues	Impact on power generation	Impact on crops
Irradiation	Although certain PV cell materials are efficient at utilizing diffuse insolation, direct insolation is generally more effective for electricity generation	The increase in the interception of solar rays by the panel will decrease the irradiation on the crops
Tilt and azimuth	- The performance of PV panels is influenced by their orientation and tilt, which affect the amount of producible energy ⁃ Maximum radiation is intercepted by the PV module with the least incident angle of sunlight	
Mutual distance	A shorter distance results in a higher panel density and consequently, greater energy production	It affects the distribution of sunlight on the underlying crops
Ambient temperature	⁃ PV efficiency diminishes at a rate of approximately 0.5% as air temperature increases by 10 °C ⁃ In a simulation study, during daytime in the peak growing seasons, the temperature of PV panels in agrivoltaic systems was found to be approximately 8.9 ± 0.2 °C cooler compared to those in conventional PV farms	The presence of crops under the panel can mitigate the effect of temperature on the PV efficiency loss
Dust accumulation	⁃ Electricity generation decreases as dust accumulation increases	Not relevant

The comparative evaluation of the candidate photovoltaic technologies was supported by several performance indicators commonly adopted in agrivoltaic studies. Among these, the land equivalent ratio (LER) was considered one of the main indicators because it quantifies the overall efficiency of the combined production of agricultural biomass and electricity relative to separate land uses (Amaducci et al. 2018) as expressed in Eq. (1).

1 $$LER=(\frac{Agricultural\;yield\;with\;agrivoltaic}{Agricultural\;yield\;with\;outagrivoltaic})+(\frac{Energy\;yield\;of\;agrivoltaic\;system}{Energy\;yield\;of\;the\;ground-mounted\;system})$$

LER values greater than 1 indicate that the integrated agrivoltaic system uses land more efficiently than separate agricultural and photovoltaic installations, whereas values lower than 1 indicate a reduction in overall land-use efficiency. Consequently, LER was adopted as one of the main indicators for comparing the proposed agrivoltaic configurations. However, although LER provides a reliable assessment of land-use efficiency, it does not account for factors such as light distribution uniformity, crop maturation time, soil degradation, or water stress. Therefore, additional performance indicators were considered to provide a more comprehensive evaluation of the proposed agrivoltaic systems (Dupraz 2023).

In addition to LER, the comparative assessment also considered the ground coverage ratio (GCR), water-saving potential, product quality, and the price-performance ratio (Ppr), as these indicators provide complementary information on the technical, agronomic, and economic performance of agrivoltaic systems.

The GCR was used to evaluate land occupation by relating the total photovoltaic module area to the available agricultural surface. Values between 0.3 and 0.4 were considered indicative of an appropriate balance between electricity production and agricultural land use. Water-saving potential was evaluated by comparing the water demand of agrivoltaic systems with that of conventional open-field cultivation. Product quality was assessed considering not only biomass yield but also qualitative characteristics reported in the literature, such as product size and nutritional properties. The economic assessment was further supported by the price-performance ratio (PPR), which compares the additional costs associated with agrivoltaic implementation with the economic benefits derived from maintaining agricultural production, as defined in Eq. (2) (Di Francia and Cupo 2023).

2 $$Ppr=\frac{P}{Pb}$$

where *P *represents the annual additional cost associated with the implementation of the agrivoltaic system compared with a conventional photovoltaic installation. *Pb *represents the economic benefit associated with preserving agricultural land use and the revenues generated by agricultural production.

### Criteria for plant selection in agrivoltaics environment

Based on the literature survey described in Section "Literature survey", candidate plant species were selected according to a set of agronomic, physiological, environmental, and economic criteria relevant to agrivoltaic applications. The evaluation considered photosynthetic efficiency, water use efficiency, crop rotation requirements, compatibility with photovoltaic technologies, and the economic value of the selected crops. These criteria were subsequently applied to identify the plant species most suitable for the proposed agrivoltaic-phytoremediation framework.

#### Photosynthetic efficiency

Photosynthetic efficiency was considered one of the primary selection criteria because the shading generated by photovoltaic panels directly influences crop growth and biomass production. Candidate species were therefore evaluated according to their ability to maintain photosynthetic activity under reduced light conditions. Particular attention was given to the carbon assimilation pathway, one of the main physiological factors affecting plant adaptation to shading (Mouhib et al. 2024). Accordingly, the preliminary selection distinguished between:

- Shade-tolerant species (C3 plants)
- Shade-intolerant species (C4 plants)

#### Water resource utilization

Water use efficiency was considered an additional selection criterion because agrivoltaic systems can reduce soil evaporation and improve water conservation through partial shading. Preference was therefore given to species characterized by high drought tolerance and efficient water use, particularly under Mediterranean climatic conditions. This criterion was considered especially relevant for environments affected by seasonal water scarcity, where agrivoltaic systems may reduce irrigation requirements by approximately 20–30% (Elamri et al. 2018).

#### Crop rotation

Crop rotation requirements were also considered during the selection process because they influence long-term soil fertility, nutrient availability, and the sustainability of agrivoltaic systems. Preference was given to species that can be integrated into diversified cropping systems, thereby reducing nutrient depletion, limiting pest and disease pressure, and improving soil quality over successive cultivation cycles. Previous agrivoltaic studies have also shown that appropriate crop rotation under photovoltaic panels can maintain stable agricultural yields while improving soil conditions under shaded environments (Barron-Gafford et al. 2016, 2019).

#### Compatibility with photovoltaic technologies

Compatibility between plant species and photovoltaic technologies was regarded as a key selection criterion. Candidate species were evaluated based on their ability to adapt to different shading levels produced by monofacial, bifacial, and semi-transparent photovoltaic systems. Special emphasis was placed on species capable of maintaining satisfactory biomass production under modified microclimatic conditions, including reduced solar radiation, lower soil temperatures, and higher soil moisture. The potential benefits of semi-transparent photovoltaic modules in mitigating shading effects while maintaining high photovoltaic performance were also considered, since these systems improve light distribution and increase light availability beneath the photovoltaic array (Gorjian et al. 2022).

#### Economic value and market

Economic aspects were also considered during the plant selection process to ensure the long-term sustainability of the proposed agrivoltaic-phytoremediation system. Preference was given to species characterized by high biomass productivity, low cultivation and management requirements, and the potential for biomass valorization through energy or industrial applications. In addition, the possibility of combining agricultural revenues with electricity production was considered an important criterion for improving the overall economic performance and resilience of the proposed agrivoltaic system (Malu et al. 2017).

## Phytoremediation and agrivoltaics integrated system

An integrated agrivoltaic-phytoremediation framework was developed to support the selection of plant species and photovoltaic configurations capable of simultaneously promoting soil remediation, biomass production, and renewable energy generation (Barron-Gafford et al. 2019). The framework was based on a joint evaluation of the biological, agronomic, environmental, and technological factors influencing the performance of both phytoremediation and agrivoltaic systems.

The study focused on the interaction between photovoltaic shading and plant physiological response, since light availability directly affects photosynthesis, biomass production, and contaminant uptake. In addition, the potential effects of photovoltaic systems on the crop microclimate, including reduced soil evaporation and improved water use efficiency, were considered because they may influence plant performance under Mediterranean environmental conditions (Barron-Gafford et al. 2025; Fagnano et al. 2024).

The selection framework also included postharvest biomass management as an additional criterion, since phytoremediation species may accumulate potentially toxic elements (PTEs) during plant growth (Liu and Tran 2021). Biomass valorization pathways reported in the literature, including thermochemical processes such as combustion, pyrolysis, and gasification, together with industrial applications involving construction materials, biocomposites, and bio-based products, were considered when evaluating the long-term sustainability and potential utilization of the selected species (Widmer et al. 2024; Tan et al. 2023; Grifoni et al. 2021; Mukherjee et al. 2025; Ryłko-Polak et al. 2022).

The literature analysis highlighted several key indicators for identifying suitable species for integrated agrivoltaic-phytoremediation systems. From a phytoremediation perspective, the most relevant criteria include tolerance to potentially toxic elements (PTEs), biomass productivity, root system development, remediation mechanisms, and the potential for biomass valorization. From an agrivoltaic perspective, additional factors such as photosynthetic pathway (C3/C4), tolerance to partial shading, water use efficiency, adaptability to Mediterranean environmental conditions, and compatibility with different photovoltaic configurations were also considered. Overall, species capable of simultaneously satisfying these complementary biological, agronomic, environmental, and economic requirements were considered the most suitable candidates for the proposed integrated system.

Based on the selection criteria described in Section "Materials and methods", three representative species were identified for the Augusta case study: *Cannabis sativa* L., *Chrysopogon zizanioides* (L.) Roberty, and *Arundo donax* L. Rather than identifying a universally optimal species, these plants were selected because they collectively satisfy the agronomic, physiological, environmental, and economic criteria established for the proposed agrivoltaic-phytoremediation framework while representing complementary remediation strategies and biomass valorization pathways.

*C. sativa* (Jurga et al. 2025) is a C3 plant capable of maintaining adequate photosynthetic efficiency even under moderate shading conditions, which are typical of agrivoltaic systems. It requires a medium water input, which can be further reduced thanks to the beneficial microclimatic effect generated by photovoltaic panels. Moreover, it has a high economic value due to its multiple industrial applications, including fiber, seeds, and secondary metabolites. Its dense root system also contributes to soil improvement, making it suitable for crop rotations.

*C. zizanioides* (Banerjee et al. 2016) represents a C4 species with high photosynthetic efficiency, notable drought resistance, and an extraordinary ability to stabilize and regenerate soils through its deep and fibrous root system. These traits make it particularly suitable for marginal areas, enabling sustainable resource management in challenging environments. Being a perennial and low-maintenance crop, it integrates well with extensive photovoltaic systems.

*A. donax* (Barbosa et al. 2015) is a C3 species well known for its hardiness and adaptability to different pedoclimatic conditions, including environments with limited water availability. It is a multipurpose crop, valuable both as a biomass source for energy and industrial applications and for environmental and phytotechnical purposes. Its capacity to thrive in marginal or degraded soils and to enhance system resilience further consolidates its suitability.

Overall, the selected species collectively satisfy the agronomic, physiological, environmental, and economic criteria established for the proposed agrivoltaic-phytoremediation framework. Their complementary tolerance to PTE contamination, response to photovoltaic shading, and biomass valorization potential provide a robust basis for evaluating integrated remediation and renewable energy production under Mediterranean environmental conditions. The following sections present the comparative assessment of their performance under PTE contamination and photovoltaic shading.

### Contaminant effect on biomass production

The first evaluation focused on the tolerance of the selected species to PTE-contaminated soils. Plant tolerance was assessed by comparing biomass production under contaminated conditions with that of uncontaminated control plants, expressed as biomass reduction relative to the control. The comparative analysis was based on experimental data reported in the literature for soils contaminated with different concentrations of potentially toxic elements (PTEs).

**Fig. 1:**
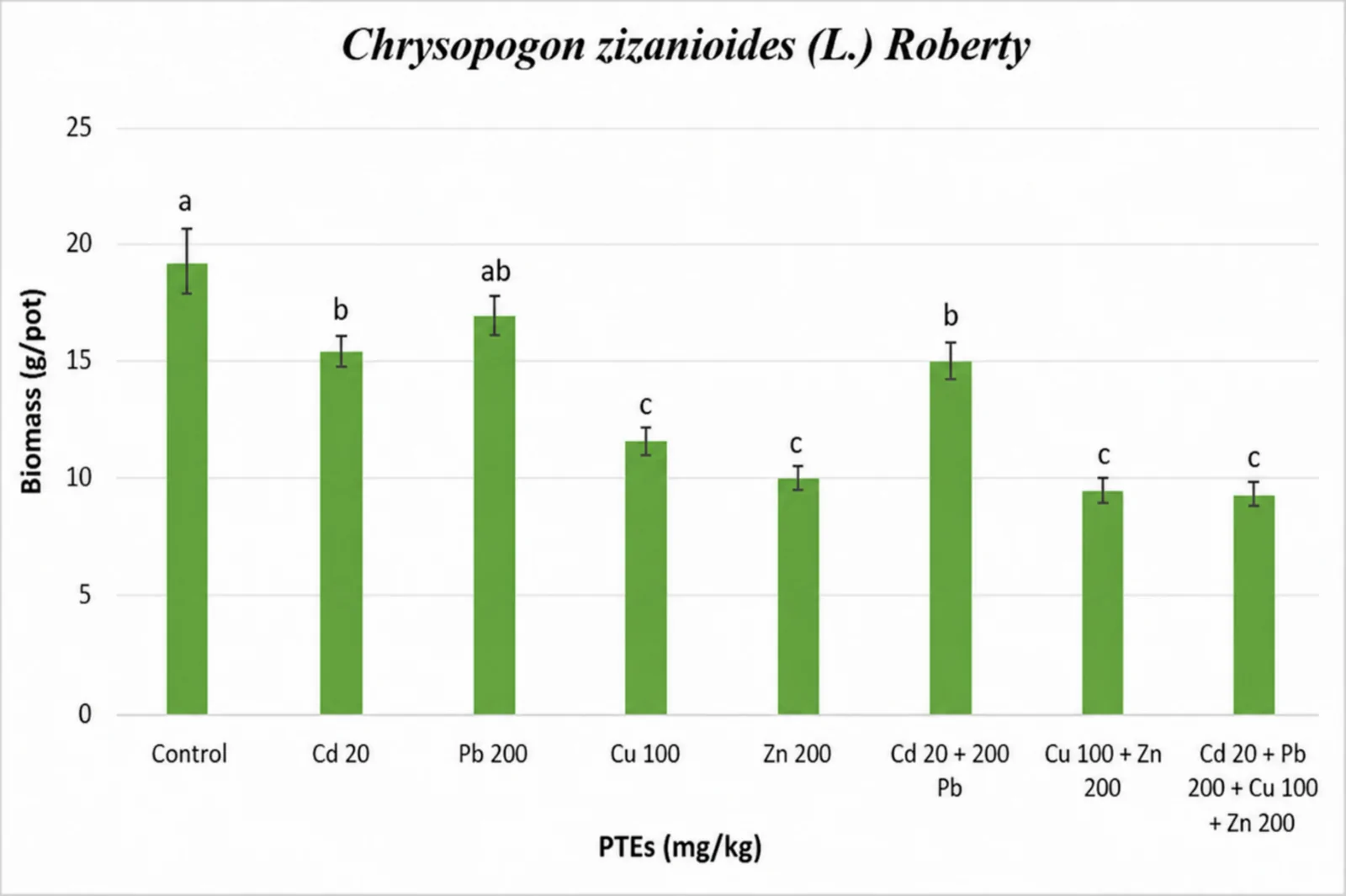
Biomass production (g pot^−1^) of *Chrysopogon zizanioides* (L.) Roberty grown in uncontaminated soil (control) and in soils contaminated with different concentrations and combinations of Cd, Pb, Cu, and Zn. Biomass values were compared with the uncontaminated control to assess the effects of potentially toxic element (PTE) contamination on plant growth and tolerance. Values are expressed as mean ± standard deviation. Different letters indicate significant differences among treatments at the 95% confidence level according to the least significant difference (LSD) test (Ng et al. 2020)

PTE contamination affected biomass production by altering physiological processes associated with plant growth (Rashid et al. 2023). Figure 1 summarizes biomass production data for *C. zizanioides* grown under different concentrations and combinations of Cd, Pb, Cu, and Zn, based on the experimental results reported by Ng et al. (2020). Biomass production was highest in the uncontaminated control and progressively decreased with increasing contaminant concentrations, although limited reductions were observed under the lowest Pb and Cd treatments, indicating good tolerance to moderate contamination levels. Biomass reductions were evaluated relative to the uncontaminated control to assess the tolerance of the species to PTE contamination.

**Fig. 2:**
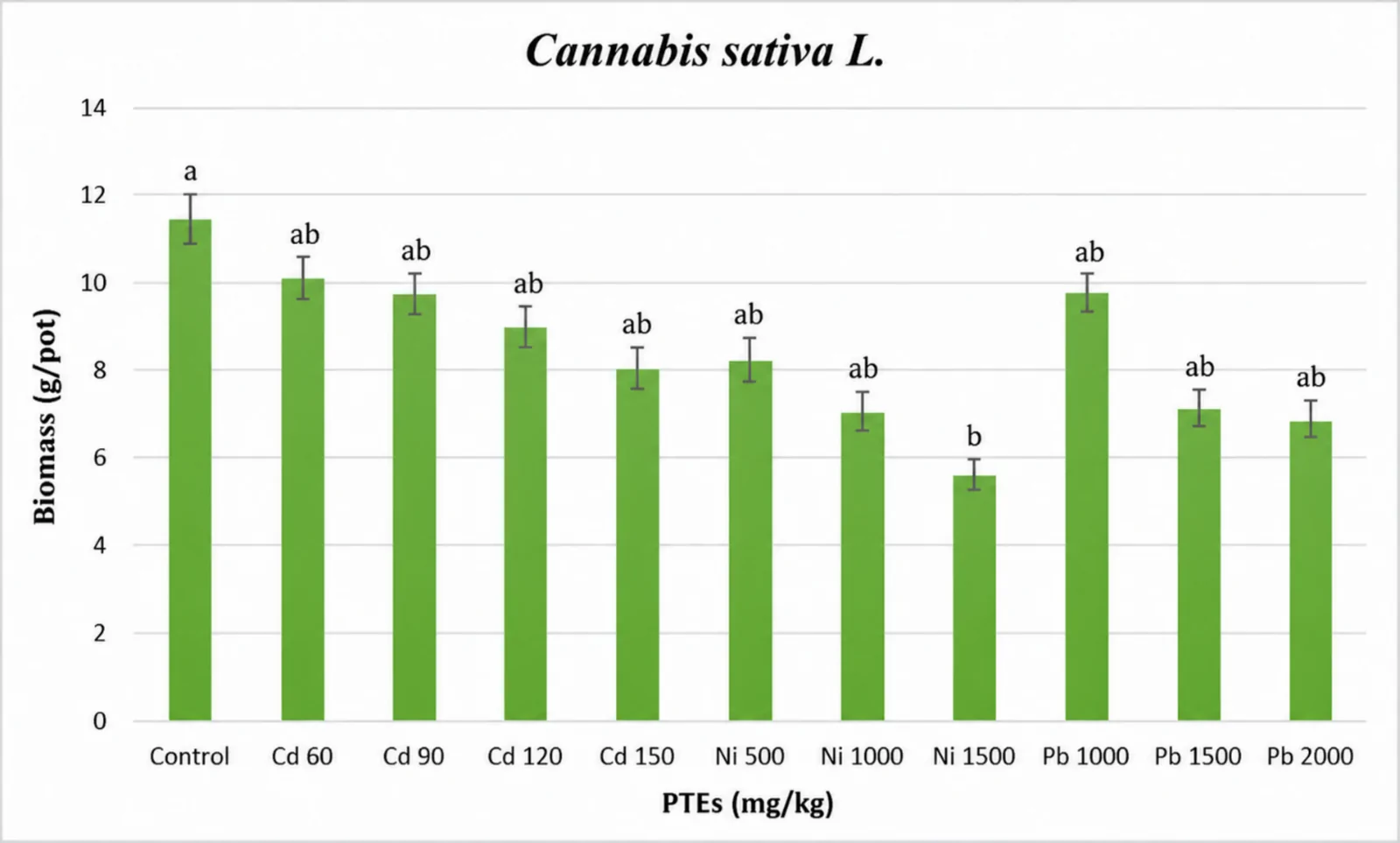
Biomass production (g pot^−1^) of *Cannabis sativa* L. grown in uncontaminated soil (control) and in soils contaminated with different concentrations of Cd, Pb, and Ni. Biomass values were compared with the uncontaminated control to assess the effects of potentially toxic element (PTE) contamination on plant growth and tolerance. Values are expressed as mean ± standard deviation. Different letters indicate significant differences among treatments according to Tukey’s honestly significant difference (HSD) test at *p* ≤ 0.05 (Testa et al. 2023)

The study by Testa et al. (2023) evaluated the late-flowering variety Futura 75 under three different levels of soil contamination from Cd, Pb, and Ni to evaluate their phytoremediation potential and the effects of the pollutants on the yield of hemp. In PTE-contaminated soil, the Futura 75 variety maintained biomass production under all tested contamination levels, although a reduction compared with the uncontaminated control was observed. The most pronounced decrease in biomass production occurred at the highest Ni concentration (Ni 1500), while Cd- and Pb-contaminated treatments showed comparatively smaller reductions. Figure 2 shows the biomass production of *C. sativa* under different Cd, Pb, and Ni contamination levels, based on data reported by Testa et al. (2023). Biomass reductions were evaluated relative to the uncontaminated control to assess plant tolerance to PTE contamination.

**Fig. 3:**
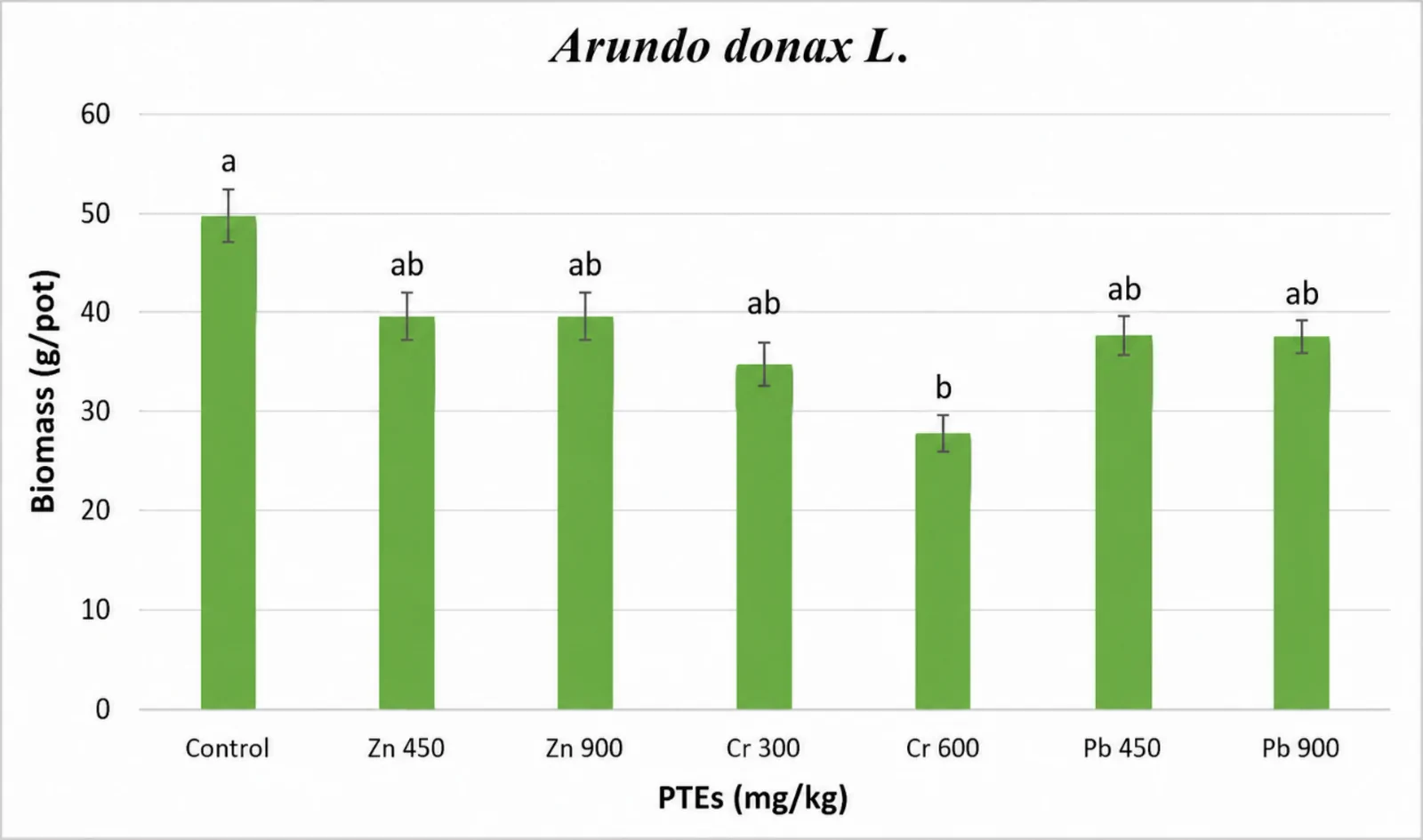
Biomass production (g pot^−1^) of *Arundo donax* L. grown in uncontaminated soil (control) and in soils contaminated with different concentrations of Zn, Cr, and Pb. Biomass values were compared with the uncontaminated control to assess the effects of potentially toxic element (PTE) contamination on plant growth and tolerance. The greatest reduction in biomass production was observed under the Cr 600 treatment. Values are expressed as mean ± standard deviation. Different letters indicate significant differences among treatments according to Tukey’s honestly significant difference (HSD) test at *p* ≤ 0.05 (Barbosa et al. 2015)

The biomass production of *A. donax* was affected by PTE contamination, with the most pronounced reduction observed at the highest Cr concentration (600 mg kg^−1^) (Fig. 3). The study also assessed the accumulation behavior of Zn, Cr, and Pb, demonstrating that *A. donax* can be considered an interesting candidate for the phytoextraction of Zn, thanks to its high capacity for metal accumulation and biomass production. Furthermore, *A. donax* genotypes proved to be well suited for the phytostabilization of PTEs contamination, reducing the leaching of PTEs and the risk of groundwater contamination (Barbosa et al. 2015).

### Shading effect on biomass production

The second evaluation focused on crop tolerance to shading induced by photovoltaic panels. Because specific studies evaluating the response of the selected species to agrivoltaic shading are still limited, the analysis was based on data extrapolated from C3 and C4 crop functional groups (Laub et al. 2021).

**Fig. 4:**
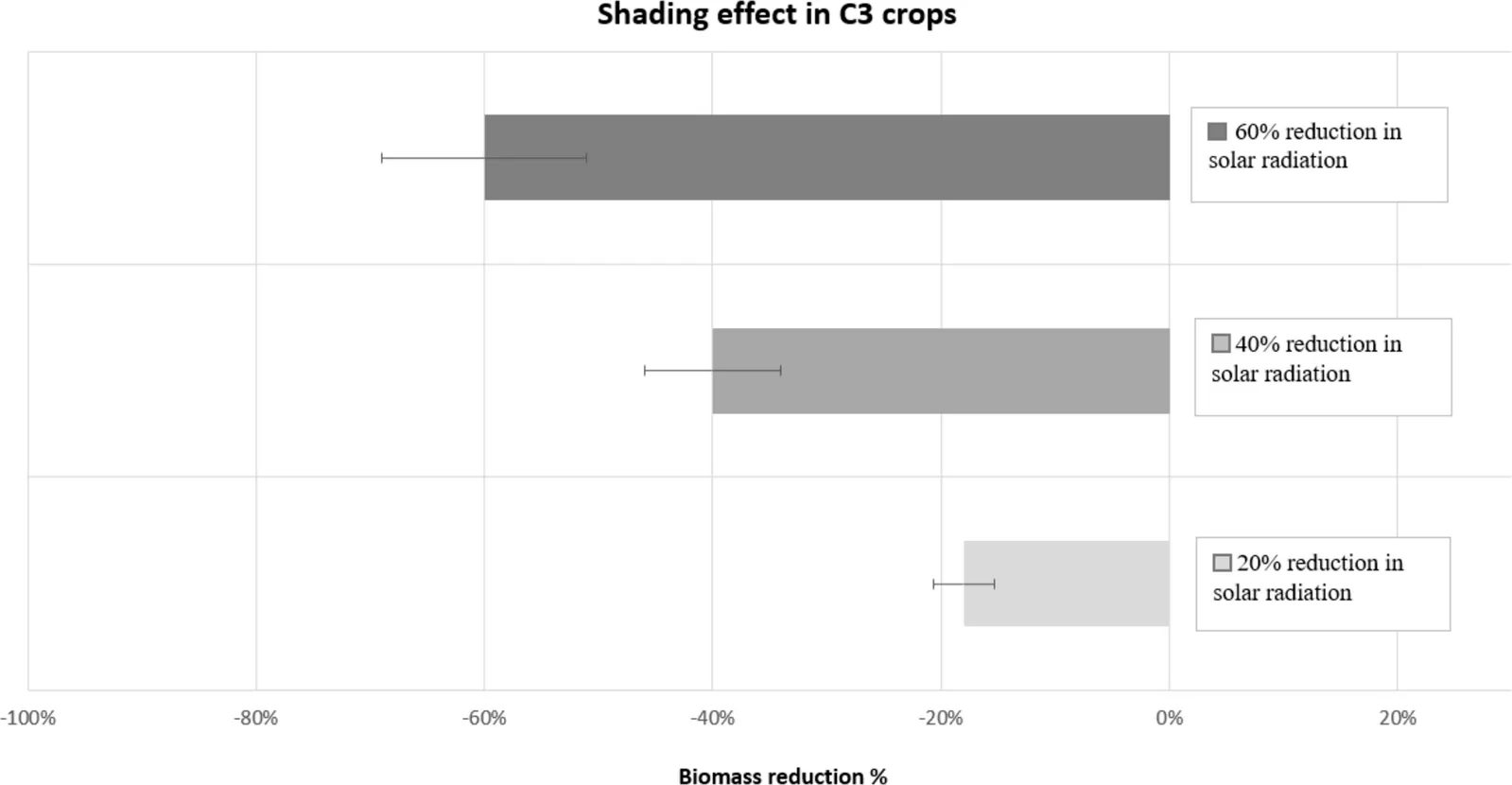
Predicted biomass reduction in C3 crops under different shading intensities (20%, 40%, and 60% reduction in solar radiation). Values represent estimates derived from the meta-analysis of (Laub et al. 2021), and error bars denote 95% confidence intervals

The quantitative analysis included data from artificial shading and intercropping/agroforestry studies to evaluate plant responses at increasing shade levels. Specific response curves have been developed as a function of solar radiation reduction, estimating crop biomass production relative to unshaded controls. The results suggest a non-linear relationship between biomass production and solar radiation reduction for all crop types. Laub et al. (2021) reported that C3 cereals exhibit a less-than-proportional yield decline up to approximately 15% reduction in solar radiation, indicating a moderate tolerance to low shading levels. Error bars represent the 95% confidence intervals of the estimated mean, indicating the statistical uncertainty associated with the predictions (Fig. 4).

**Fig. 5:**
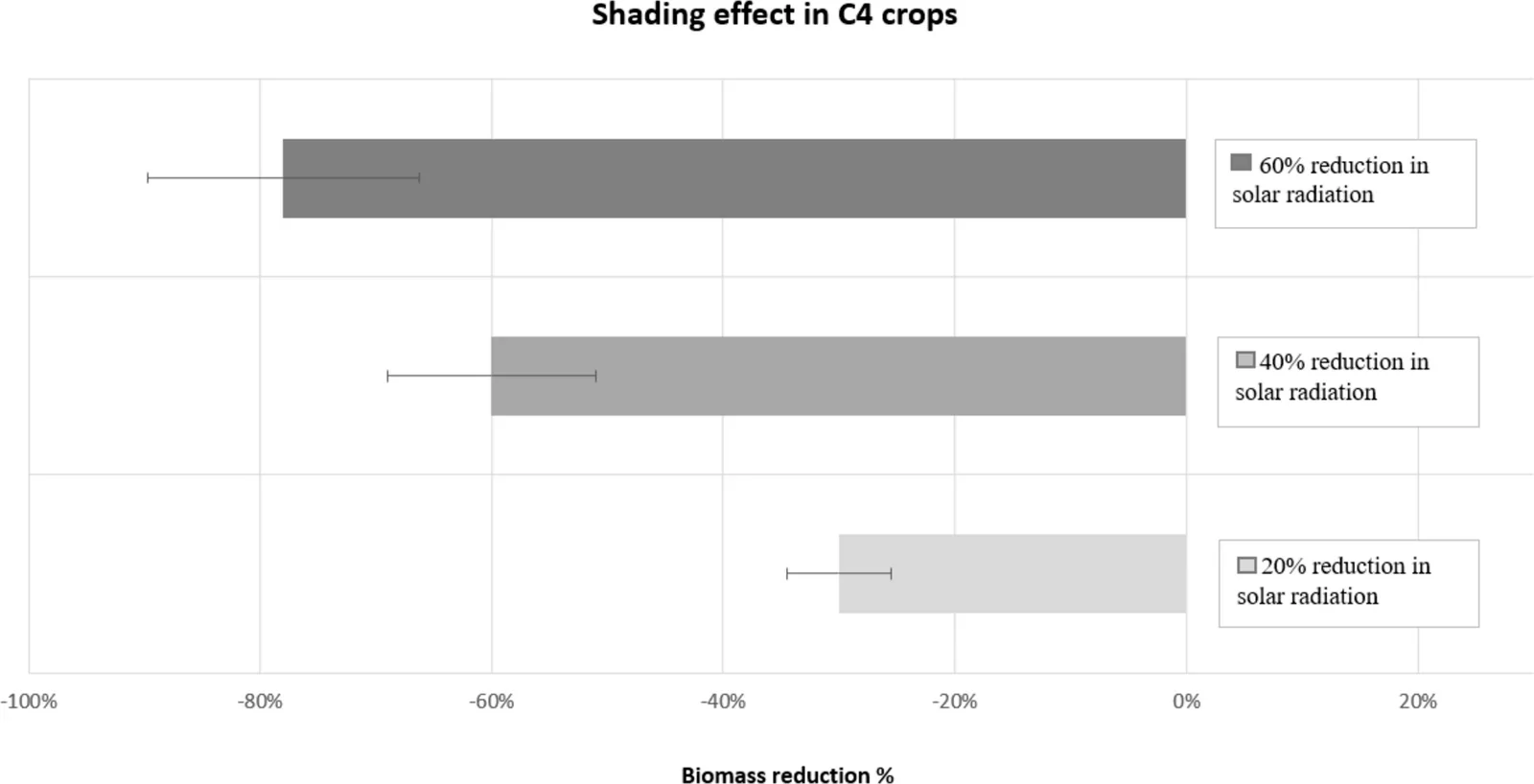
Predicted biomass reduction in C4 crops under different shading intensities (20%, 40%, and 60% reduction in solar radiation). Values represent estimates derived from the meta-analysis of (Laub et al. 2021). Error bars indicate 95% confidence intervals of the estimated mean, reflecting the uncertainty associated with the meta-regression model. Wider confidence intervals denote higher variability and lower data availability at specific RSR levels

In contrast to C3 crops, C4 species exhibited greater sensitivity to increasing shading levels, with biomass reductions becoming more pronounced as solar radiation decreased (Fig. 5).

## Case study: Augusta (Italy)

This section applies the agrivoltaic-phytoremediation framework developed in the previous sections to the Augusta case study. The objective is to evaluate the proposed approach through three representative agrivoltaic layouts, assessing renewable energy generation, biomass production, and techno-economic performance under the environmental conditions of the study area. Augusta (Sicily, Italy) was selected because of its severe environmental contamination and the availability of reliable scientific data supporting the quantitative assessment.

The industrial area of Augusta, located in eastern Sicily (37° 13′ 49″ N, 15° 13′ 10″ E), is one of the main industrial hubs of the Augusta–Priolo–Melilli petrochemical district (Romano et al. 2021).

The area hosts numerous refineries and chemical and petrochemical industries whose activities have resulted in severe environmental contamination. Industrial emissions have released high concentrations of potentially toxic elements (Pb, Cd, Hg, and As), together with organic pollutants such as polycyclic aromatic hydrocarbons (PAHs), PCBs, and dioxins, into the surrounding soil and water.

These factors have caused severe environmental degradation, leading to the designation of the area as a Site of National Interest (SIN) for remediation. For soil remediation, Articles 242 and 242-bis of Legislative Decree 152/2006 are applicable, which regulate site characterization and intervention procedures. These procedures must be aligned with the Sicilian Regional Remediation Plan, which establishes methodologies and intervention priorities.

The soils of the Augusta area are heavily degraded from a pedological perspective. Contamination with PTEs and toxic substances has reduced land fertility, increasing risks to both human health and the ecosystem (Ausili et al. 2015). The soil is predominantly calcareous, typical of Sicilian seaside regions, and in industrial areas, its structural deterioration has been observed (ISPRA 2011). In some areas, clay-rich soil layers promote water stagnation during intense rainfall, hindering natural drainage and further complicating environmental remediation. Moreover, the proximity to the sea and the lack of adequate vegetation increase the risk of erosion and desertification, phenomena that further exacerbate the overall picture of soil pollution.

The climate of the Augusta area is Mediterranean, characterized by long, hot, dry summers and mild, rainy winters. Average summer temperatures range between 25 and 30 °C, but during heat waves, they can exceed 35 °C. In winter, temperatures rarely drop below 5 °C. Precipitation is concentrated in autumn and winter, with an annual average of about 500–600 mm. Given the dry summer conditions, careful water management is essential for both agricultural production and phytoremediation activities. The winds, which blow predominantly from the north and south, contribute to the dispersion of atmospheric pollutants produced by industrial activities. In particular, the Sirocco, a hot wind from the Sahara, can worsen summer drought conditions, further aggravating the issue of water supply. Relative humidity is generally high due to the proximity to the sea, a factor that can affect both the local microclimate and the dynamics of pollutant dispersion in the atmosphere and soil. The sea surrounding Augusta Bay has undergone significant degradation, with negative impacts on both marine flora and fauna. Moreover, the presence of the sea influences soil salinity, an important factor to consider in remediation and phytoremediation activities, as some crops may be affected by the high salt concentration in the soil.

Following the assessment of crop tolerance to PTE contamination and photovoltaic shading, the final pairing between plant species and photovoltaic technologies was proposed by matching the physiological, agronomic, and economic characteristics of each species with the light environment and operational features of the different photovoltaic configurations. Rather than maximizing a single performance indicator, the objective was to identify representative integrated solutions capable of balancing phytoremediation efficiency, biomass production, renewable electricity generation, and biomass valorization under the environmental conditions of the Augusta site.

Accordingly, *Arundo donax L.* was proposed for integration with monofacial photovoltaic modules because of its high biomass productivity, robust adaptability to the pedoclimatic conditions of the Augusta area, and greater tolerance to partial shading than the other selected C3 species. The high energy yield and commercial maturity of conventional monofacial photovoltaic technology make this configuration particularly suitable for projects prioritizing renewable electricity generation while maintaining satisfactory biomass production. *Chrysopogon zizanioides *(L.) Roberty was associated with vertical bifacial photovoltaic modules because their more homogeneous light distribution and reduced direct shading are better suited to the higher irradiance requirements of this C4 species while fully exploiting its excellent drought tolerance, high water use efficiency, and deep root system. Finally, *Cannabis sativa *L. was paired with semi-transparent photovoltaic modules because their higher light transmittance helps mitigate shading effects, allowing this moderately shade-tolerant C3 species to maintain satisfactory biomass productivity while preserving its phytoremediation potential. Overall, these proposed pairings reflect the complementary characteristics of both the plant species and the photovoltaic technologies, providing representative configurations for evaluating integrated agrivoltaic-phytoremediation systems under Mediterranean environmental conditions.

The main agronomic characteristics and phytoremediation potential of the three proposed species are summarized in Table 5 (Truong and Thai Danh 2015; Tang et al. 2017; Scordia et al. 2011).

**Table 5:** Agronomic characteristics and phytoremediation potential of *Chrysopogon zizanioides* (L.) Roberty, *Cannabis sativa* L., and *Arundo donax* L., including biomass yield, cultivation parameters, root depth, and absorbed potentially toxic elements (PTEs)

Plant	Biomass yield	Seeding	Planting density (m)	Harvest	Depth roots (m)	Absorbed PTEs	Reference
*Chrysopogon zizanioides* (L.) Roberty	50–100 t ha^−1^ y^−1^	March/April	0.3 × 0.5	July/August	3–4	As, Cd, Cu, Cr, Pb, Zn, Hg	Dudai et al. (2006)
*Cannabis sativa* L.	15–25 t ha^−1^ y^−1^	March/April	0.6 × 0.1	July/August	2–2.5	Cd, Cu, Cr, Pb, Zn, Ni	Visković et al. (2023)
*Arundo donax* L.	30–40 t ha^−1^ y^−1^	March/April	1 × 1	September/October	0.8–1.4	Cd, Cr, Cu, Ni, Pb	Danelli et al. (2020)

Beyond their phytoremediation potential and adaptation to the environmental conditions of the Augusta site, the three proposed species were selected because they collectively satisfy the agronomic, physiological, and economic criteria defined in Section "Materials and methods". Their complementary characteristics, including photosynthetic pathway, water use efficiency, shading tolerance, biomass productivity, and biomass valorization potential, provide a comprehensive basis for evaluating integrated agrivoltaic-phytoremediation systems. The comparative assessment of these characteristics is reported in Table 6.

**Table 6:** Comparative assessment of the selected plant species for agrivoltaic applications based on photosynthetic pathway, water-use characteristics, and shading tolerance

Selection criteria	*Arundo donax* L	*Cannabis sativa* L	*Chrysopogon zizanioides* (L.) Roberty
Photosynthetic efficiency	C3	C3	C4
Water resource utilization	High water efficiency, drought tolerant	Requires moderate irrigation	Highly efficient in water use, drought tolerant
Compatibility with photovoltaics	Shade tolerant	Moderately shade tolerant	Low shade tolerance
Crop rotation	Contributes to the recovery of marginal lands	Improves weed and pest management	Reduces erosion, enhances aeration, and improves the soil's capacity to retain humidity
Economic value and market	High-value biomass for energy production	Various applications in the industrial materials	Used in the cosmetic industry
Reference	(Danelli et al. 2020)	(Visković et al. 2023)	(Dudai et al. 2006)

### Economic assessment based on biomass valorization

From an economic perspective, integrating phytoremediation with agrivoltaic systems can generate dual benefits by combining the progressive remediation of PTE-contaminated soils with biomass valorization for energy production.

In this context, the three selected species (*C. sativa*, *A. donax*, and *C. zizanioides*) represent promising candidates because they combine documented tolerance to contaminants with high biomass production potential.

The harvested biomass can be valorized through thermochemical gasification, producing syngas that can subsequently be converted into electricity. For an illustrative estimate, recent literature data were considered, including the lower heating value (LHV) of lignocellulosic biomass (≈18 MJ kg^−1^), gasification efficiency (≈60%), syngas calorific value (≈5–6 MJ Nm^−3^), and electrical conversion efficiency through gas engines (≈30%) (Segers et al. 2024) (Sher et al. 2025). Assuming an average European electricity price reported by Eurostat (2024) of approximately €0.215 kWh^−1^, the corresponding gross revenues per hectare were estimated (Table 7). Biomass yields were derived from local-scale experimental data and case studies, assuming average values of 75 t ha^−1^ year^−1^ for *C. zizanioides*, 20 t ha^−1^ year^−1^ for *C. sativa*, and 35 t ha^−1^ year^−1^ for *A. donax*. Stress effects related to contaminants were assumed following a precautionary approach: *C. sativa* may experience biomass reductions up to 50% under high Ni concentrations (1500 mg kg^−1^), *A. donax* up to 40% for Cr > 600 mg kg^−1^, and *C. zizanioides* up to 50% under critical Zn, Pb, and Cd concentrations. Regarding shading, differentiated effects are expected among the configurations considered, depending on the type of photovoltaic module adopted. In the case of *A. donax* cultivated under tilted monofacial panels, a reduction in photosynthetically active radiation (PAR) between 25 and 30% is assumed. For *C. sativa* combined with semi-transparent panels, the estimated average shading ranges between 20 and 25%, whereas for *C. zizanioides* grown under vertical bifacial panels, a more limited reduction of 10–15% is assumed, due to lower direct interception and enhanced radiation diffusion. In all cases, the reported shading percentages represent estimates derived from the plant configurations selected and simulated in this study (module geometry, orientation, and row arrangement) and are therefore used as parametric inputs for yield assessment. These PAR reductions were conservatively translated into biomass losses of 25–30% for *C. sativa*, 20–25% for *A. donax*, and 15–20% for *C. zizanioides*, consistent with the different physiological responses of C3 and C4 species under partial shading conditions.

**Table 7:** Estimated biomass production, gross revenues, management costs, and net revenues for the selected crops under baseline, shading, and contamination scenarios

Crop	Scenario	Biomass (t ha^−1^ y^−1^)	Gross revenue (€ ha^−1^ y^−1^)	Management cost (€ ha^−1^ y^−1^)	Net revenue (€ ha^−1^ y^−1^)
***Chrysopogon zizanioides*** (L.) Roberty	Baseline	75.0	14,512	5,750	8,762
	Shading 15%	60.0	11,610	4,880	6,730
	Contamination	37.5	7,256	3,575	3,681
***Cannabis sativa*** L.	Baseline	20.0	3,900	1,535	2,365
	Shading 25%	14.0	2,730	1,187	1,543
	Contamination	10.0	1,950	955	995
***Arundo donax*** L.	Baseline	35.0	6,772	2,680	4,092
	Shading 20%	26.3	5,079	2,173	2,906
	Contamination	21.0	4,063	1,868	2,195

In addition to gross revenues, biomass management costs must be considered. Mechanical harvesting entails an average cost of €20–30 t^−1^ (Spinelli et al. 2005), transport to gasification facilities ranges between €5–15 t^−1^ depending on distance (Scarlat et al. 2015), while pretreatment and chipping are estimated at €10–15 t^−1^ (Scarlat et al. 2015). Furthermore, the disposal of contaminated ashes, requiring specific treatments or landfill in authorized facilities, may cost €100–200 t^−1^ of ash produced (Sher et al. 2025). Considering an average ash yield of 5–8% of the initial dry mass, this burden can significantly reduce the net profitability of the system. When outsourced, harvesting of *C. sativa* and the biannual pruning of *A. donax* and *C. zizanioides* incur additional costs estimated at €100–150 t^−1^ of dry biomass, thereby further reducing net revenues (Dyjakon a García-Galindo 2019).

Based on these assumptions, gross economic revenues from syngas-based electricity production range from €8,762 ha^−1^ year^−1^ for *C. zizanioides* under optimal conditions to €995 ha^−1^ year^−1^ for *C. sativa* under severe stress (Table 7). However, when including biomass management and ash disposal costs, net revenues can decline by 30–50%, highlighting the importance of optimizing logistics and integrating alternative biomass uses (e.g., biochar, biocomposites) to improve economic sustainability. Despite these limitations, this example provides a useful indication of the strategic potential of the phytoremediation–agrivoltaic approach for valorizing marginal lands.

### Integrated system options in the selected case study

Based on the selection criteria established in Section "Materials and methods" and the representative species–photovoltaic pairings proposed for the Augusta case study, three integrated agrivoltaic configurations were developed and evaluated through PVsyst simulations. The objective was to assess the technical feasibility and energy performance of each configuration by optimizing the photovoltaic layout while ensuring compatibility with the physiological, agronomic, and economic characteristics of the selected crops. Simulations were performed for a 1-hectare area under the environmental conditions of Augusta, allowing a comparative evaluation of electricity generation and biomass production for the proposed integrated systems.

In the considered agrivoltaic context, the selected crops—including *A. donax*, *C. sativa*, and* C. zizanioides—*were assumed to be managed through carefully planned agronomic practices, which are essential to ensure compatibility between the crops and the photovoltaic structures. To ensure adequate light exposure, facilitate mechanized pruning and biomass harvesting, and minimize interference with the panels, a recommended panel height was set at 4–4.5 m above ground (Dupraz et al. 2011; Marrou et al. 2013). This configuration provides ample vertical space for tall-growing species, enhancing air circulation and creating microclimatic conditions favorable to crop development.

Agronomic management included periodic pruning to control plant height and density, reducing the risk of shading and contact with the panels while optimizing the quantity and quality of biomass produced. These strategies not only support vigorous crop growth without compromising panel functionality but also facilitate continuous contaminant uptake, thereby enhancing phytoremediation efficiency and strengthening the synergy between renewable energy production and environmental remediation. The combination of targeted agronomic management, precise panel height design, and optimal row spacing allows for an effective balance between agricultural and energy production, enhancing the overall sustainability and profitability of the agrivoltaic system.

#### Integrated system: *Chrysopogon zizanioides* (L.) Roberty-bifacial PV

A bifacial photovoltaic panel is a solar module capable of capturing solar radiation on both sides. When installed vertically, bifacial modules improve light distribution between panel rows while simultaneously generating electricity from both faces. The increase in energy yield depends on several factors, including ground reflectivity (albedo), ground clearance, and module orientation (Muehleisen et al. 2021). The distance between rows significantly affects the distribution of PAR, and crop yield can be almost halved when the distance between mutual rows changes from 20 to 5 m. By examining the contributions of LER for a specific year (i.e., contributions to crop yield and electricity production), it is evident that the optimal distance between rows varies according to the crop (Campana et al. 2021). The optimization results indicate that LER alone cannot be used as the primary criterion for designing agrivoltaic systems. Additional performance indicators should be considered to better evaluate the trade-offs between crop productivity and electricity generation. LER maximization drastically reduces electricity production, which could compromise the investment in the PV system, or could lead to crop failure (Campana et al. 2021).

Previous studies have shown that the implementation of an agrivoltaic system can lead to a LER greater than 1.2, meaning a productivity increase of more than 20% for the same land compared to monoculture. Values greater than 1.5 have been reported by Trommsdorff et al. (2021), while values greater than 1.2 have been reported by Amaducci et al. (2018). Previous optimization studies have shown that the optimal azimuth angle is generally close to − 40°, whereas the optimal row spacing typically ranges between 8 and 10 m. The optimal panel orientation depends on the project’s objectives. South-facing configurations maximize electricity production but require crops with greater tolerance to shading. In contrast, vertical east–west configurations improve light distribution between panel rows, making them more suitable for agricultural production while maintaining satisfactory energy generation.

In this agrivoltaic context, *C. zizanioides* was selected, especially for its ability to adapt to challenging soils and tolerate partial shading conditions. Its strong resilience to unfavorable environmental conditions, such as drought and degraded soils, makes it particularly suitable for complex contexts like the Augusta area. Previous studies have demonstrated that *C. zizanioides* is highly tolerant of saline and alkaline soils, as well as a wide range of soil pH conditions, making it particularly suitable for agrivoltaic applications in degraded environments (Truong et al. 2008). The combination of bifacial photovoltaic modules and *C. zizanioides* is particularly suitable because the vertical configuration improves light distribution between panel rows, helping to mitigate shading effects on this C4 species (Truong and Thai Danh 2015).

**Fig. 6:**
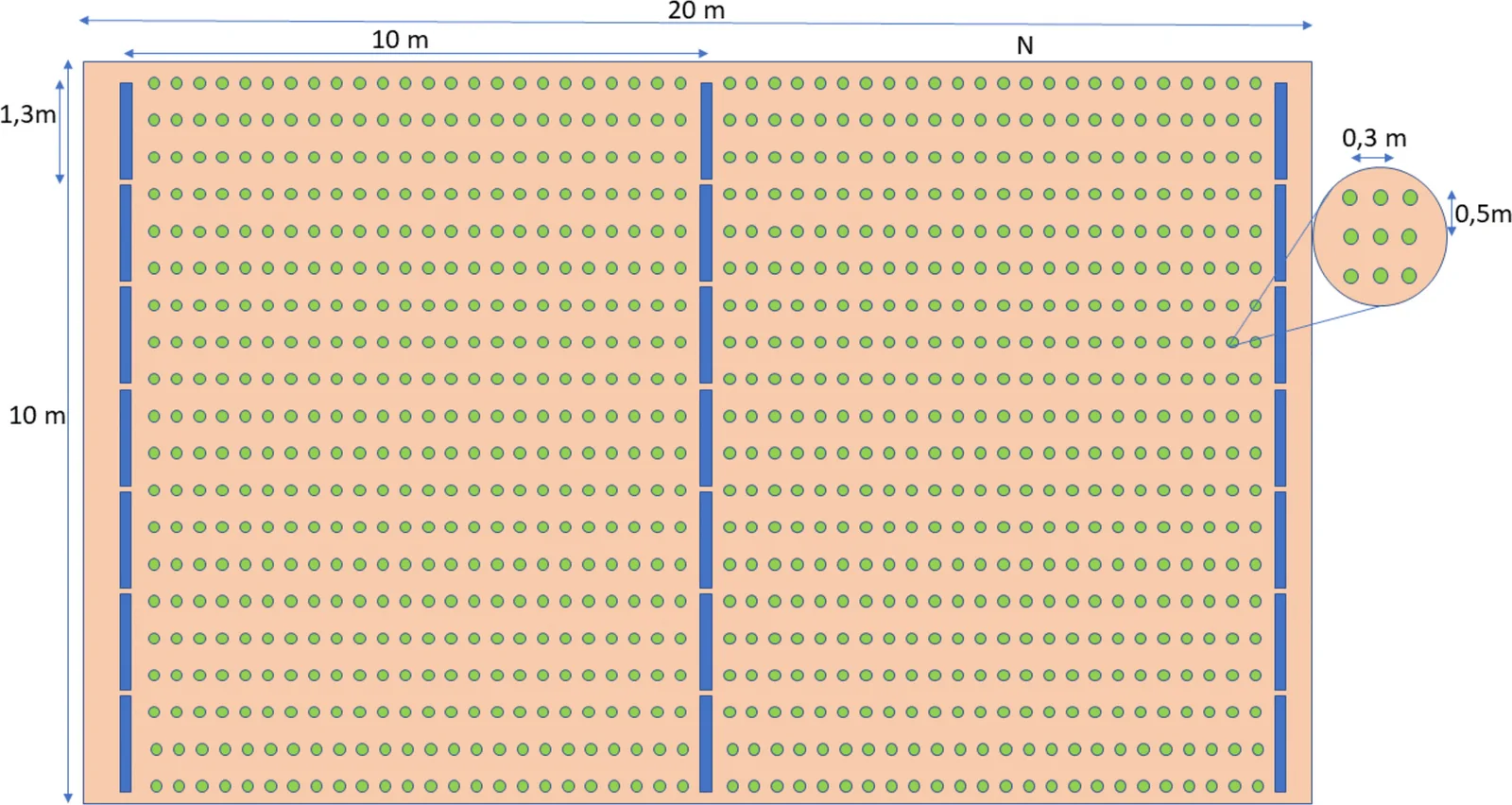
Layout of the proposed bifacial agrivoltaic system integrated with *Chrysopogon zizanioides* (L.) Roberty. The figure shows the spatial arrangement of the vertically mounted bifacial photovoltaic rows and the cultivated area within the simulated 1-hectare layout, including the adopted row spacing and panel orientation

A PVsyst simulation was performed for a 1-hectare area using vertical bifacial photovoltaic modules with a nominal power of 440 Wp. The modules were arranged in an east–west orientation with a row spacing of 10 m, resulting in a total of 11 rows and 1008 panels (Fig. 6). The simulated annual electricity production was conservatively reduced by 20% to account for overall photovoltaic system losses (Bergin et al. 2017) (Table 8).

**Table 8:** Technical specifications and estimated annual electricity production of the simulated bifacial agrivoltaic system proposed for the Augusta case study. The table summarizes the main design parameters adopted in the PVsyst simulation, including the photovoltaic technology, number of modules, occupied surface area, row spacing, module power, tilt angle, azimuth, and the corresponding annual electricity production

Technology panel	N.modules	Surface (m^2^)	Row space (m)	Wp	Tilt	Azimuth	Annual production (kWh)
Bifacial	1008	10,000	10	440	90°	− 40°	297.987

#### Integrated system: *Arundo donax* L.-monofacial PV

Among the photovoltaic technologies considered, conventional monofacial modules provide the highest electricity production because they maximize the direct conversion of incident solar radiation (Sugianto 2020). However, row spacing and panel orientation play a fundamental role, ensuring a balance between energy production and the growth of the underlying crops.

Several studies have investigated the influence of row spacing and panel orientation on agrivoltaic system performance. For instance, a north–south panel orientation with wider row spacing (10 m or more) improves light distribution and reduces shading on the underlying crops, while still maintaining high energy efficiency of the system. This configuration is particularly advantageous in environments with high solar irradiance, as controlled shading and reduced evapotranspiration support crop growth during the hotter months (Younas et al. 2019).

In this agrivoltaic context, *A. donax* was selected because of its high biomass productivity, adaptability to Augusta’s pedoclimatic conditions, and greater tolerance to partial shading compared with other candidate species. Its robustness and adaptability enable satisfactory biomass production even under partial shading. Scordia et al. (2011) demonstrated the remarkable adaptability of *A. donax* to Mediterranean environments, supporting its suitability for agrivoltaic applications. Additionally, *A. donax* is known for its resistance to saline soils, making it particularly suitable for marginal or lower-quality soils that might hinder some other species’ growth. Additional studies have shown that *A. donax* tolerates high salinity through physiological mechanisms that limit sodium accumulation in leaf tissues (Cosentino et al. 2014).

**Fig. 7:**
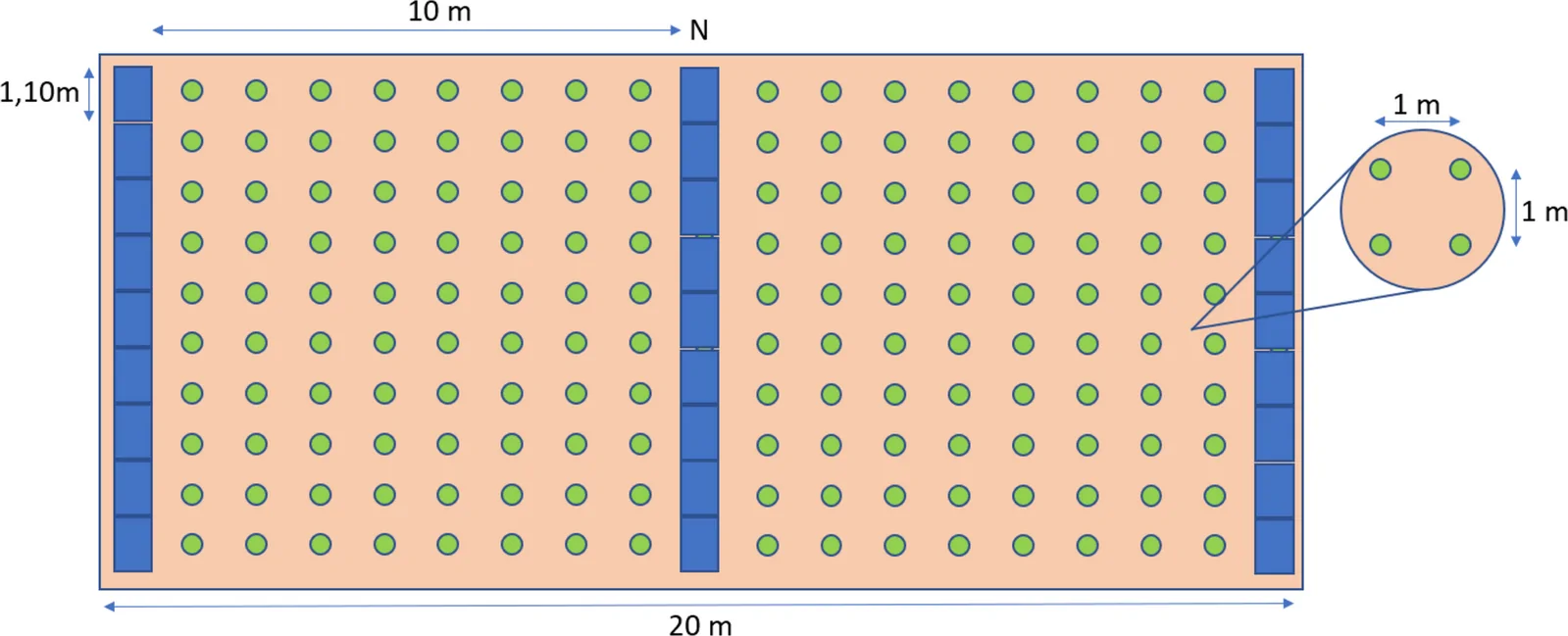
Layout of the proposed monofacial agrivoltaic system integrated with *Arundo donax* L. The figure shows the spatial arrangement of the monofacial photovoltaic rows and the cultivated area within the simulated 1-hectare layout, including the adopted row spacing and panel orientation

To limit shading, the system was designed with a 10-m row spacing between panel rows, ensuring adequate light availability for crop growth. A PVsyst simulation was performed for a 1-hectare area using monofacial photovoltaic modules with a nominal power of 450 Wp. Panel rows were oriented in the N-S direction with a distance between them of 10 m for a total of 11 rows with 1026 panels (Fig. 7). The estimated energy production was reduced by 20% to account for overall photovoltaic system losses (Bergin et al. 2017) (Table 9).

**Table 9:** Technical specifications and estimated annual electricity production of the simulated monofacial agrivoltaic system proposed for the Augusta case study. The table summarizes the main design parameters adopted in the PVsyst simulation, including the photovoltaic technology, number of modules, occupied surface area, row spacing, module power, tilt angle, azimuth, and the corresponding annual electricity production

Technology panel	N.modules	Surface(m^2^)	Row space (m)	Wp	Tilt	Azimuth	Annual production (kWh)
Monofacial	1026	10,000	10	450	31°	0°	568.660

#### Integrated system: *Cannabis sativa* L.-semitransparent PV

Semi-transparent photovoltaic technologies (STPV) expand the application of photovoltaics in agrivoltaic systems by increasing light availability for the underlying crops. STPV developments incorporate traditional thin-film solar cell technologies (amorphous-Si, CdTe, and CIGS) and newer technologies (organic, perovskite, and dye-sensitized cells). Significant advancements have been made both in power conversion efficiency (PCE) and semi-transparency of these devices (Kumar et al. 2023). Their partial light transmittance allows sufficient solar radiation to reach the crops below, reducing biomass losses while maintaining electricity production. This characteristic makes them particularly suitable for moderately shade-tolerant species such as *C. sativa*.

In this agrivoltaic context, *C. sativa* was selected because the higher light transmittance provided by semi-transparent photovoltaic modules helps mitigate shading effects while maintaining satisfactory biomass productivity under agrivoltaic conditions. Moreover, the pedoclimatic conditions of the Augusta area are well suited to its cultivation (Campiglia et al. 2017). Although *C. sativa* tolerates moderate shading, maintaining adequate light availability is essential to minimize biomass losses and preserve its phytoremediation potential.

Previous studies have highlighted the adaptability of *C. sativa* to different soil types and Mediterranean climatic conditions (Tang et al. 2017), supporting its suitability for cultivation in the Augusta area.

**Fig. 8:**
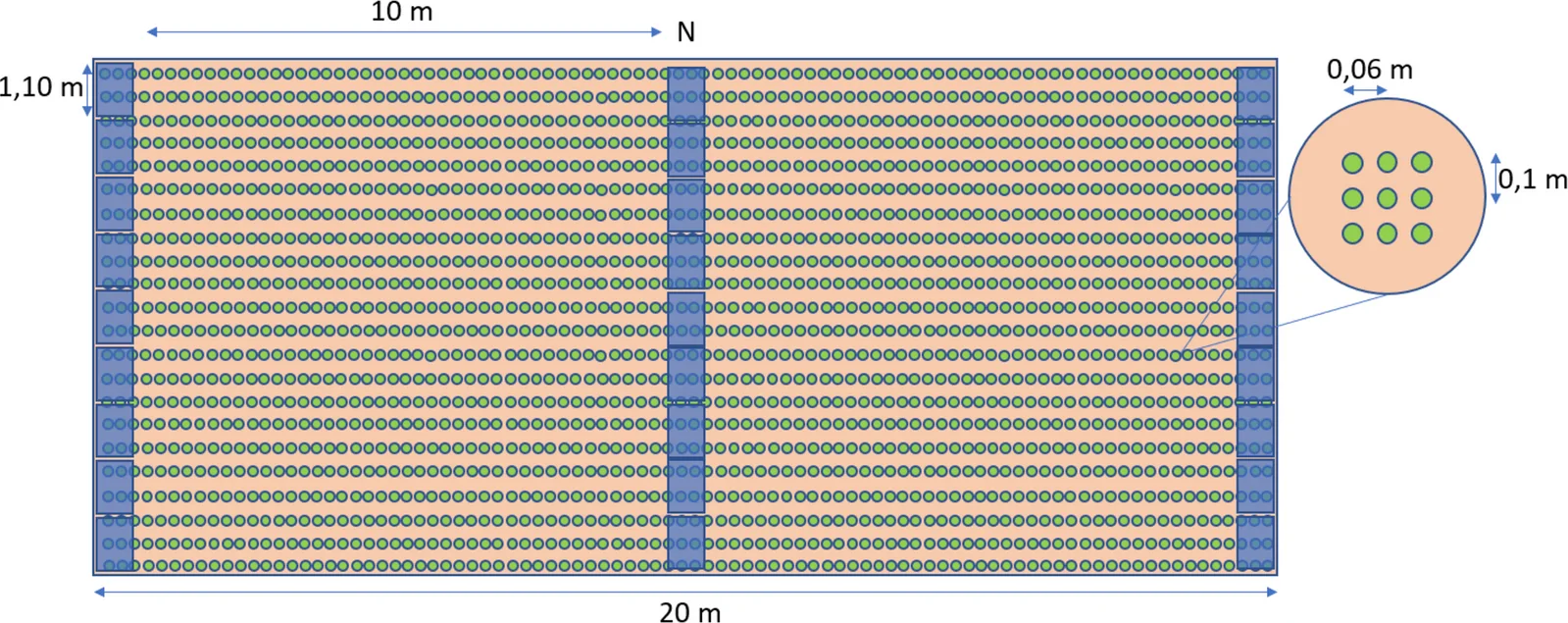
Layout of the proposed semi-transparent agrivoltaic system integrated with Cannabis sativa L. for the Augusta case study. The figure illustrates the spatial arrangement of the semi-transparent photovoltaic rows and the crop distribution within the simulated 1-hectare layout. The photovoltaic modules are arranged in north–south-oriented rows spaced 10 m apart to maximize light transmission while maintaining satisfactory biomass production and renewable electricity generation

A PVsyst simulation was performed for a 1-hectare area using semi-transparent photovoltaic modules with a nominal power of 440 Wp. The modules were arranged in a north–south orientation with a row spacing of 10 m, resulting in a total of 11 rows and 528 panels (Fig. 8). The simulated annual electricity production was conservatively reduced by 20% to account for overall photovoltaic system losses (Bergin et al. 2017) (Table 10).

**Table 10:** Technical specifications and estimated annual electricity production of the simulated semi-transparent agrivoltaic system proposed for the Augusta case study. The table summarizes the main design parameters adopted in the PVsyst simulation, including the photovoltaic technology, number of modules, occupied surface area, row spacing, module power, tilt angle, azimuth, and the corresponding annual electricity production

Technology panel	N.modules	Surface (m^2^)	Row space (m)	Wp	Tilt	Azimuth	Annual production (kWh)
Semitransparent	528	10,000	10	440	31°	0°	285.638

The selection of photovoltaic technology should be guided by the primary objectives of the agrivoltaic system. Monofacial modules maximize electricity production and bifacial modules provide the best compromise between electricity generation and biomass production, whereas semi-transparent modules are particularly suitable for maintaining biomass productivity in moderately shade-tolerant crops such as *C. sativa*, despite their lower electricity yield.

In the agrivoltaic-phytoremediation context, the choice should be carefully considered. Given the typically slow remediation process, energy production generally represents the primary objective; however, some field trials are still required to determine whether the selected crops can adapt to the simultaneous exposure to two major stress factors: shading and the presence of soil contaminants.

## Conclusion

In this study, crop tolerance was evaluated in terms of biomass reduction under PTE contamination and partial shading. In addition, techno-economic estimates were developed, and PVsyst simulations were performed to quantify the electricity production of the proposed agrivoltaic configurations. However, studies simultaneously addressing both PTE contamination and photovoltaic shading within the same agrivoltaic framework remain extremely limited. For this reason, crop species capable of tolerating both stress factors were identified to assess their potential application in highly contaminated and arid environments typical of many industrial areas. The Augusta–Priolo industrial area was therefore adopted as a case study to demonstrate the technical feasibility of integrating phytoremediation and agrivoltaic systems. Three representative agrivoltaic configurations combining different photovoltaic technologies and phytoremediation species were proposed and comparatively evaluated for the Augusta case study.

Regarding *Arundo donax* L., the results indicate that biomass production is significantly reduced mainly under high contaminant concentrations, with yield losses up to 40% in soils contaminated with more than 600 mg kg^−1^ of Cr. Under the contamination scenario considered, biomass decreases to 21.0 t ha^−1^ year^−1^, with a corresponding net revenue of €2,195 ha^−1^ year^−1^. Under shading conditions (− 20%), biomass declines to 26.3 t ha^−1^ year^−1^, with a net revenue of €2,906 ha^−1^ year^−1^. The species confirms good phytoextraction potential for Zn, Cr, and Pb; however, its well-known invasiveness, due to its extensive rhizome system, represents a relevant limitation for large-scale applications, requiring containment strategies or the evaluation of alternative species with comparable potential.

For *Cannabis sativa* L., the findings highlight good tolerance to PTEs, with marked biomass reductions occurring only under high Ni concentrations (up to − 50%). In this scenario, biomass reaches 10.0 t ha^−1^ year^−1^, with a net revenue of €995 ha^−1^ year^−1^. Under shading (− 25%), biomass decreases to 14.0 t ha^−1^ year^−1^, corresponding to a net revenue of €1,543 ha^−1^ year^−1^. Despite these reductions, the species maintains positive profitability, confirming its suitability for agrivoltaic systems aimed at soil remediation.

For *Chrysopogon zizanioides* (L.) Roberty, biomass production is high under optimal conditions (75 t ha^−1^ year^−1^) but more sensitive to the presence of metals in the soil. Under critical contamination levels (Zn, Pb, Cd), biomass is reduced by 50%, reaching 37.5 t ha^−1^ year^−1^, with a net revenue of €3,681 ha^−1^ year^−1^. Under shading conditions (− 15%), biomass amounts to 60.0 t ha^−1^ year^−1^, with a net revenue of €6,730 ha^−1^ year^−1^. Despite its higher susceptibility to contaminants, its substantial biomass production makes this species economically attractive.

From an agrivoltaic perspective, the optimal configuration depends on the objectives of the system. The monofacial/*A. donax* configuration provides the highest electricity production (568,660 kWh year^−1^), whereas the bifacial/*C. zizanioides* (297,987 kWh year^−1^) and semi-transparent/*C. sativa* (285,638 kWh year^−1^) configurations offer a better balance between renewable energy generation and biomass production. Considering the estimated biomass reductions under shading (− 25% for *C. sativa*, − 20% for *A. donax*, and − 15% for *C. zizanioides*), the bifacial/*C. zizanioides *configuration represents the most balanced solution among the three configurations evaluated in this study, combining satisfactory energy production with the highest net revenue among the three proposed agrivoltaic systems. In contrast, *C. sativa* showed greater tolerance to PTE contamination but lower overall economic returns under the simulated conditions.

Future research should focus on validating the proposed agrivoltaic configurations under field conditions and on assessing the combined effects of long-term PTE contamination and photovoltaic shading on crop performance and phytoremediation efficiency.

## Data Availability

The data shown in the article are provided below for greater convenience and clarity.
